# How do existing HIV-specific instruments measure up? Evaluating the ability of instruments to describe disability experienced by adults living with HIV

**DOI:** 10.1186/1477-7525-8-88

**Published:** 2010-08-19

**Authors:** Kelly K O'Brien, Ahmed M Bayoumi, Carol Strike, Nancy L Young, Kenneth King, Aileen M Davis

**Affiliations:** 1 Department of Health Policy, Management and Evaluation, University of Toronto, Toronto, Ontario, Canada; 2 Centre for Research on Inner City Health, The Keenan Research Centre in the Li Ka Shing Knowledge Institute, St. Michael's Hospital, 30 Bond Street, Toronto, Ontario, M5B 1W8, Canada; 3 School of Rehabilitation Science, McMaster University, 1400 Main Street West, Room 403, Hamilton, Ontario, L8S 1C7, Canada; 4 Department of Medicine, University of Toronto, Toronto, Ontario, Canada; 5 Dalla Lana School of Public Health, University of Toronto, 155 College Street, Health Science Building, 6th floor, Toronto, Ontario, M5T 3M7, Canada; 6 Centre for Addiction and Mental Health, Toronto, Ontario, Canada; 7 School of Rural and Northern Health, Laurentian University, 935 Ramsey Lake Road, Sudbury, Ontario, P3E 2C6, Canada; 8 Canadian Working Group on HIV and Rehabilitation, 1240 Bay Street, Suite 600, Toronto, Ontario, M5R 2A7, Canada; 9 Division of Health Care and Outcomes Research and Arthritis and Community Research and Evaluation Unit, Toronto Western Research Institute, 399 Bathurst Street - MP11-322, Toronto, Ontario, M5T 2S8, Canada

## Abstract

**Background:**

Despite the multitude of health challenges faced by adults living with HIV, we know of no HIV-specific instrument developed for the purpose of describing the health-related consequences of HIV, a concept known as disability. In a previous phase of research, adults living with HIV conceptualized disability as symptoms/impairments, difficulties carrying out day-to-day activities, challenges to social inclusion, and uncertainty that may fluctuate on a daily basis and over the course of living with HIV. In this paper, we describe the extent to which existing HIV-specific health-status instruments capture the experience of disability for adults living with HIV.

**Methods:**

We searched databases from 1980 to 2006 for English language, HIV-specific, self-reported questionnaires consisting of at least two items that were tested for reliability and validity. We then conducted a content analysis to assess how well existing questionnaires describe disability as defined by the *Episodic Disability Framework*, a framework that conceptualizes this experience from the perspective of adults living with HIV. We matched items of the instruments with categories of the framework to evaluate the extent to which the instruments capture major dimensions of disability in the framework.

**Results:**

We reviewed 4274 abstracts, of which 30 instruments met the inclusion criteria and were retrieved. Of the four major dimensions of disability, symptoms/impairments were included in all 30 instruments, difficulties with day-to-day activities in 16, challenges to social inclusion in 16, and uncertainty in 9. Seven instruments contained at least 1 item from all 4 dimensions of disability (breadth) however, the comprehensiveness with which the dimensions were represented (depth) varied among the instruments.

**Conclusions:**

In general, symptoms/impairments and difficulties carrying out day-to-day activities were the disability dimensions characterized in greatest depth while uncertainty and challenges to social inclusion were less well represented. Although none of the instruments described the full breadth and depth of disability as conceptualized by the *Episodic Disability Framework*, they provide a foundation from which to build a measure of disability for adults living with HIV.

## Background

With longer survival, HIV-positive individuals are facing an increasing variety of health-related consequences and symptoms related to HIV infection, associated treatment, and concurrent health conditions [1-11]. Together, these experiences may be conceptualized as disability. We developed a conceptual framework of disability from the perspective of adults living with HIV. In the *Episodic Disability Framework*, adults living with HIV defined disability as symptoms/impairments, difficulties carrying out day-to-day activities, challenges to social inclusion, and uncertainty that may fluctuate on a daily basis and over the entire course living with HIV [12,13].

Developing programs or interventions to address HIV-related disability mandates the development of a measurement instrument. A patient-reported disability questionnaire might assess the impact of disability for both clinical care and societal level decision making. To date, we know of no instrument developed for the purpose of describing HIV-specific disability. Related instruments, such as functional status and quality of life measures, capture some aspects of disability but may not be comprehensive when considering the range of health-related consequences of HIV [14-19]. Generic disability instruments may not capture population-specific disability experiences [20-23]. The purpose of this research was to evaluate the extent to which HIV-specific health status instruments capture disability experienced by adults living with HIV using the *Episodic Disability Framework*.

## Methods

### The Episodic Disability Framework

In a prior phase of research, we developed a conceptual framework of disability from the perspective of adults living with HIV. Specifically, we conducted four focus groups and 15 face-to-face interviews with 38 adults living with HIV, asking individuals to describe their health-related challenges, the physical, social and psychological areas of their life affected, and the impact of these challenges on their overall health. The resulting *Episodic Disability Framework *conceptualizes disability as multi-dimensional and episodic in nature. The framework is comprised of three main components: 1) dimensions of disability, 2) contextual factors that may exacerbate or alleviate disability, and 3) triggers or life events that may initiate a major or momentous episode for adults living with HIV. Details of this framework were previously published [12,13].

### Instruments: Search Strategy and Inclusion Criteria

**Figure 1:**
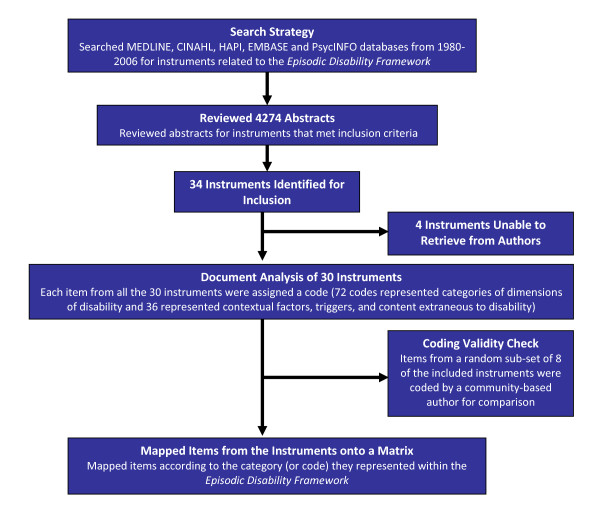
**Overview of Content Analysis Methodology: **An overview of the content analysis methodology including the search strategy, abstract review, document analysis of included instruments, validity check, and mapping of items from the instruments according to the category (or code) they represented in the *Episodic Disability Framework.*

To identify measures related to disability, we systematically searched the health and psychology literature for instruments that capture elements of the disability experience for adults living with HIV (Figure 1). We searched the following databases for articles published between 1980 and March 2006: MEDLINE, CINAHL, HAPI, EMBASE, and PsycINFO. Subject headings included exploded terms for HIV, HIV infections, health status indicators, quality of life, disability evaluation, behaviour and behaviour mechanisms, activities of daily living, psychiatric status rating scales, data collection, work, socioeconomic factors, signs and symptoms, mental disorders, uncertainty, culture, family, social environment, social isolation, socialization, sociometric techniques, religion, spiritual therapies, and stigma. Slight modifications of this strategy were made for each database. We reviewed abstracts yielded from the search for instruments relevant to disability. If it was unclear from the abstract whether an instrument was applicable, we pulled the full article for review. We also searched reference lists from pertinent articles for potentially relevant instruments.

We included instruments that were published in English, were HIV-specific self-reported questionnaires including at least two items, and had been tested for reliability and validity. We excluded instruments that measured constructs un-related to the four dimensions of disability in the *Episodic Disability Framework*. When we were uncertain whether to include an instrument or if the instrument was not published within the article, we requested further information from study authors.

### Analysis

**Figure 2:**
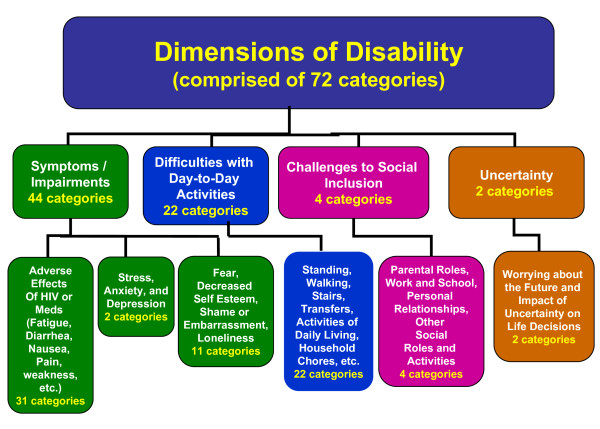
**Episodic Disability Framework: **The four dimensions of disability in the *Episodic Disability Framework *and the number of categories that represent each dimension used for the content analysis.

We analyzed instruments using content analysis, a qualitative method in which pre-defined categories of text are matched against each other and used to compare documents [24]. We compared each instrument against the *Episodic Disability Framework *[12] We evaluated the instruments against the dimensions of disability in the framework [12] (Figure 2). These dimensions were classified into 10 high-level categories and 72 detailed sub-categories. For example, an item about fatigue received a high-level category of "symptom/impairment" and a sub-category of "fatigue/decreased energy level." We created new sub-categories for instrument items that did not match a pre-identified classification. These new sub-categories represented contextual factors or triggers of disability or items beyond the scope of the framework. See Additional File 1 for a detailed overview of categories.

One author categorized all instruments. To assess validity, we assessed agreement between this categorization and that of a community-based author who categorized eight randomly selected instruments. We calculated percent agreement for each instrument by dividing the number of items categorized identically by the total number of items in the instrument. We determined percent agreement for detailed sub-categories, high-level categories, and dimensions of disability. The two raters reconciled any differences by consensus.

We mapped items from the instruments onto a matrix according to the category that they represented within the disability framework. An instrument with greater representation of the dimensions of disability in this matrix was determined *a priori *to possess a greater ability to describe the construct of disability for adults living with HIV. We classified an instrument as having breadth if it contained at least one item from each of the four disability dimensions. We classified an instrument as having depth (for each dimension) if it contained items which corresponded to all pre-specified categories in a given dimension.

## Results

We reviewed 4274 abstracts, of which 34 instruments met the inclusion criteria. Instruments were excluded because they were un-related to the *Episodic Disability Framework, *were measures of adherence to medications, attitudes towards death, internal locus of control, attitudes towards health providers, quality of care, satisfaction, utility indices, disclosure, knowledge about HIV/AIDS, sexual and risk behaviour. Of the 34 instruments identified for inclusion, 30 were retrieved (Table 1). We were unable to retrieve four instruments after three attempts to contact the authors [25-28].

**Table 1:** Characteristics of Instruments Included in the Content Analysis (n = 30 instruments)

Instrument	Authors	Construct Measured^	Year Developed	Number of Items
Body Image Scale [41]	Martinez et al	Body Image	2005	12

Assessment of Body Change and Diarrhea Scale (ACBD) [42]	Guaraldi et al	Body Image	2006	27

HIV Diarrhea Questionnaire [43]	Mertz et al	Diarrhea	1995	14

HIV-Related Fatigue Scale [44]	Barroso & Lynn	Fatigue	2000	56

Health-Related Quality of Life Scale (HIV-QOL) [18]	Cleary et al	HRQL/QOL	1993	46

AIDS Health Assessment Questionnaire (AIDS-HAQ) [45]	Lubeck & Fries	HRQL/QOL	1994	55

Functional Assessment of HIV Infection (FAHI) [29,46]	Cella & Peterman	HRQL/QOL	1997	47

HIV Overview of Problems-Evaluation System (HOPES) [30,47]	Ganz & Schag	HRQL/QOL	1992	177

HIV/AIDS Targeted QOL (HAT-QOL) [31,48]	Holmes & Shea	HRQL/QOL	1999	35

HIV Patient Assessed Report of Status and Experience (HIV-PARSE) [49]	Bozzette et al	HRQL/QOL	1989	144

HIV QOL Questionnaire (HIV-QL31) [32]	Leplege et al	HRQL/QOL	1997	31

Medical Outcomes Survey HIV Health Survey (MOS-HIV) [50,51]	Wu et al	HRQL/QOL	1997	35

Multidimensional QOL Questionnaire for HIV/AIDS (MQoL-HIV) [33]	Avis et al	HRQL/QOL	1994	40

World Health Organization QOL HIV Instrument (WHOQOL-HIV) [34,52,53]	Fang, O'Connell & WHO HIV/AIDS Quality of Life Group	HRQL/QOL	2002	120

General Health Self Assessment [54]	Lenderking et al	HRQL/QOL	1997	50

Living with HIV Scale[55]	Holzemer et al	HRQL/QOL	1998	32

HIV Cost and Services Utilization Tool [56]	Hays et al	HRQL/QOL	1998	31

AIDS Clinical Trials Group (ACTG Outcomes SF-21) [57]	AIDS Clinical Trials Group Outcomes Committee	HRQL/QOL	1999	21

Existential Loneliness Questionnaire [58]	Mayers et al	Loneliness	2002	22

Mental Adjustment to HIV Scale (MAHIVS) [59]	Ross et al	Psychological Adjustment	1994	40

HIV/AIDS Stress Scale [35]	Pakenham & Rinaldis	Stress	2002	29

HIV Stressor Scale [60]	Thompson et al	Stress	1996	25

Physical Symptoms of Illness Scale [27]	Nokes et al	Symptoms	1994	15

HIV Symptom Index (Justice) [61]	Justice et al	Symptoms	1998	20

Sign and Symptom Checklist for HIV (SSC-HIV) [62]	Holzemer et al	Symptoms	1999	26

Riverside Symptom Checklist [63]	Burgess et al	Symptoms	1993	28

Revised Sign and Symptom Checklist for HIV (SSC-HIVrev) [64]	Holzemer et al	Symptoms	2001	72

HIV Symptom Index (Whalen) [65]	Whalen	Symptoms	1994	12

Self-Report Slowness Scale (SRSS) [66]	Lopez et al	Symptoms	1998	11

Impact of Weight Loss Scale [67]	Wagner & Rabkin	Weight Loss	1999	9

^Construct measured as defined by the author.

HRQL = health-related quality of life; QOL = quality of life

### Description of Instruments

The included instruments were developed between 1989 and 2006, 19 of which were published after 1996 when triple drug combination antiretroviral therapy started to be used widely. The number of items in the instruments ranged from nine in the Impact of Weight Loss Scale to 177 in the HIV Overview of Problems-Evaluation System (HOPES). Instruments measured nine different constructs as identified by authors, the majority of which included health-related quality of life/quality of life (HRQL/QOL) (n = 14 instruments), followed by symptoms (n = 7), body image (n = 2), stress (n = 2), fatigue (n = 1), diarrhea (n = 1), loneliness (n = 1), psychological adjustment (n = 1), and impact of weight loss (n = 1) (Table 1).

### Document Analysis

There were 108 possible categories to which an item could be assigned for the document analysis, 72 of which represented categories within the four dimensions of disability within the *Episodic Disability Framework *(Figure 2). An additional 36 categories were generated; 15 of which represented contextual factors (n = 12) and triggers (n = 3) of disability within the framework and 21 that went beyond the scope of the *Episodic Disability Framework *(see Additional File 1 for a detailed overview of categories).

Our validity check demonstrated that agreement for the sub-set of eight instruments varied depending on the level to which the items were categorized. At the most detailed category level (108 possible categories), agreement ranged from 52% in the HIV Quality of Life Questionnaire (HIV-QL31) to 79% in the Functional Assessment of HIV Infection (FAHI) Questionnaire. At the high-level categorization (10 possible categories), agreement ranged from 61% in the HIV-QL31 to 85% in the FAHI Questionnaire. At the dimension of disability level (4 possible categories), we achieved 100% agreement for all eight instruments.

### Breadth and Depth of Disability in Instruments

Of the four major dimensions in the *Episodic Disability Framework*, symptoms/impairments were included in all 30 instruments, difficulties with day-to-day activities in 16, challenges to social inclusion in 16, and uncertainty in 9 (Table 2). Seven instruments demonstrated breadth, that is, they measured some part of all 4 dimensions of disability [29-35]. The number of items in these instruments ranged from 29 (HIV/AIDS Stress Scale) to 177 (HOPES). Authors classified six of the seven scales as HRQL/QOL instruments [29-34], and the other, a stress scale [35] (Table 1).

**Table 2:** Breadth and Depth of Disability in Instruments

	Dimensions of Disability				Breadth and Depth of Disability	
**Instrument**	**Symptoms/Impairment/44 categories**	**Difficulties with Day-to-Day Activities/22 categories**	**Challenges to Social Inclusion/4 categories**	**Uncertainty/2 categories**	**Breadth (Yes/No)**	**Depth (Yes/No)**

Body Image Scale	5				No	No

Assessment of Body Change and Diarrhea Scale (ACBD)	9			1	No	No

HIV Diarrhea Questionnaire	2				No	No

HIV-Related Fatigue Scale	4	10	2		No	No

Health-Related Quality of Life Scale (HIV-QOL)	18	8			No	No

AIDS Health Assessment Questionnaire (AIDS-HAQ)	2	12			No	Yes

Functional Assessment of HIV Infection (FAHI)	12	1	4	1	Yes	Yes

HIV Overview of Problems-Evaluation System (HOPES)	25	10	4	1	Yes	Yes

HIV/AIDS Targeted QOL (HAT-QOL)	7	1	2	2	Yes	Yes

HIV Patient Assessed Report of Status and Experience (HIV-PARSE)	21	12	3		No	Yes

HIV QOL Questionnaire (HIV-QL-31)	9	6	1	1	Yes	No

Medical Outcomes Survey HIV Health Survey (MOS-HIV)	8	6	2		No	No

Multidimensional QOL Questionnaire for HIV/AIDS (MQoL-HIV)	8	8	1	1	Yes	No

World Health Organization QOL HIV Instrument (WHOQOL-HIV)	11	6	3	1	Yes	Yes

General Health Self Assessment	16	7	2		No	No

Living with HIV Scale	9				No	Yes

HIV Cost and Services Utilization Tool	4	10	2		No	No

AIDS Clinical Trials Group (ACTG Outcomes SF-31)	5	6	2		No	No

Existential Loneliness Questionnaire	4		1		No	No

Mental Adjustment to HIV Scale (MAHIVS)	4			1	No	No

HIV/AIDS Stress Scale	8	5	3	1	Yes	No

HIV Stressor Scale	1		2		No	No

Physical Symptoms of Illness Scale	12				No	No

HIV Symptom Index (Justice)	18				No	No

Sign and Symptom Checklist for HIV (SSC-HIV)	13				No	No

Riverside Symptom Checklist	18				No	No

Revised Sign and Symptom Checklist for HIV (SSC-HIVrev)	27				No	Yes

HIV Symptom Index (Whalen)	12				No	No

Self-Report Slowness Scale (SRSS)	1	9			No	No

Impact of Weight Loss Scale	3		2		No	No

Number of categories of disability represented for each dimension within existing HIV-specific instruments (in alphabetical order based on construct measured). Breadth of disability is defined as an instrument having at least 1 item (or category) represented in each of the four disability dimensions. Depth of disability is defined as having all possible categories represented in a given dimension.

No instrument captured all of the dimensions of disability comprehensively. The depth in which the dimensions of disability were represented varied among the instruments (Table 2). We highlight eight instruments that most comprehensively represented each of the 4 dimensions of disability.

The HOPES instrument most broadly captured symptoms/impairments representing 25 categories, eight of which related to stress, anxiety and depression and emotional challenges. The Revised Sign and Symptom Checklist (SSC-HIVrev) captured 27 categories, of which two addressed stress, anxiety and depression, and emotional challenges. Alternatively, the World Health Organization's Quality of Life HIV Instrument (WHOQOL-HIV) and Living with HIV Scale were the most comprehensive at capturing symptoms/impairments that specifically related to stress, anxiety and depression, and emotional challenges with seven and eight categories, respectively, but possessed fewer categories that represented physical symptoms/impairments (4 categories in the WHOQOL-HIV and 1 category in the Living with HIV Scale).

For difficulties with day-to-day activities, the AIDS Health Assessment Questionnaire (AIDS-HAQ) and HIV Patient Assessed Report of Status and Experience (HIV-PARSE) each captured the most depth in this dimension (Table 2). Items captured a range of daily activities, some of which included walking, stair negotiation, activities of daily living, and household chores, all of which were sub-categories in the *Episodic Disability Framework*.

The FAHI and the HOPES represented all categories of challenges to social inclusion. The most common element of social inclusion missing from the other instruments that represented this dimension related to items that captured the challenges related to fulfilling parental roles (Table 2).

Uncertainty was less well represented by the instruments. The HIV/AIDS Targeted Quality of Life Scale (HAT-QOL) was the most comprehensive capturing both categories from this dimension. The remaining eight instruments (out of nine) that represented the dimension of uncertainty all captured one category comprised of items that addressed worrying about the future, but did not address the impact uncertainty has on making life decisions (Table 2).

Five of the eight comprehensive instruments were developed from 1996 onwards (Table 1). These instruments frequently captured challenges to social inclusion and uncertainty. Four instruments (FAHI, HOPES, HAT-QOL and WHOQOL-HIV) demonstrated both breadth and depth. The HOPES was the only instrument that demonstrated depth in more than one dimension (symptoms/impairments and challenges to social inclusion).

## Discussion

No existing HIV-specific health instrument fully captured both the breadth and depth of disability as conceptualized from the perspective of adults living with HIV in the *Episodic Disability Framework*. Several possible reasons explain this finding. First, these instruments were not developed to measure disability. Accordingly, we did not expect these instruments to fully capture the breadth and depth of disability. Second, disability is a new and emerging construct in the context of HIV. Recent development of the *Episodic Disability Framework *identified features of disability that were not considered a component of disablement in earlier generic disability frameworks, which explains why uncertainty was less represented among these older measures. Third, many instruments were developed prior to the advent of combination antiretroviral therapy and may not address associated new complexities relating to adverse effects, stigma and disclosure, access issues, and uncertainty about long term outcomes of treatment. Fourth, many of the quality of life instruments we studied were modified from existing generic instruments (e.g. MOS-HIV) or disease-specific instruments in other contexts such as cancer (e.g. HOPES). Such instruments might not capture disablement unique to adults living with HIV, such as issues related to returning to work. Fifth, a greater number of items did not always translate into a greater ability for an instrument to capture disability. For example, while two instruments appeared to possess breadth or depth at capturing dimensions of disability, they were lengthy comprised of more than 140 items (HIV-PARSE and HOPES scale). They demonstrated redundancy within a given category raising questions about feasibility for use of these measures in a clinical setting. Altogether, it is not surprising that existing instruments do not fully address the spectrum of disability for adults living with HIV. Nevertheless, analyses of these questionnaires may serve as a foundation from which to build a disability instrument.

A measure of disability that corresponds to dimensions of the *Episodic Disability Framework *could be developed by pooling items from existing instruments into a new one for adults living with HIV. For example, most items from existing instruments represented symptoms/impairments from the framework. This was not surprising given 16 of the 30 instruments were developed for the purpose of either measuring a combination of symptoms (n = 7) or a specific symptom/impairment (n = 9). Difficulties with day-to-day activities also were well captured by the instruments, commonly represented in instruments originally developed to measure symptoms/impairments and HRQL/QOL. The depth in which these two dimensions were represented provide a comprehensive group of existing items from which to pool together and formulate domains of symptoms/impairments and difficulties with day-to-day activities of a future disability measure.

Challenges to social inclusion and uncertainty were less well represented in the instruments. Since the introduction of combination antiretroviral therapy, there has been a shift to consider the broader health-related consequences that adults living with HIV might experience and specifically disability is becoming increasingly important to consider in the context of HIV [36]. Issues related to labour force and income support and worrying about the unpredictable and episodic nature of HIV are examples of types of disability faced by adults living longer with HIV. Accordingly, newer instruments appeared to more closely capture these two disability dimensions in the *Episodic Disability Framework *and may be a source from which to draw existing items for a new measure. Nevertheless, generation of new items will likely be required to fully capture these dimensions.

Results from this content analysis may be used to build a new HIV-specific disability questionnaire. For each of the disability dimensions we may identify instruments that most comprehensively cover a dimension with the least amount of item redundancy. Items from the next most comprehensive instruments may be used to fill any remaining gaps in existing categories. Categories not represented by any existing items would require item generation and could be done in consultation with adults living with HIV. This process may yield a collection of items that comprehensively represent each of the four disability dimensions that could be combined to comprise a new measure of HIV-disability. Once developed, measurement properties of this questionnaire including sensibility, validity, reliability and responsiveness could be assessed with adults living with HIV.

Our study has limitations. We excluded generic instruments or instruments developed for use with other illness populations in order to focus on describing disability specifically from the experience of adults living with HIV. We also excluded questionnaires that addressed other components of the *Episodic Disability Framework *(contextual factors and triggers of disability). However, these instruments may possess content that relates to the dimensions of disability experienced by adults living with HIV. We only cross-validated eight instruments in the document analysis from which low levels of agreement at the sub-category level were initially attained. This was likely due to the large number of categories that an item could be assigned. New questionnaires also have been published since March 2006 and are not captured in this analysis. We performed an updated search from 2006-July 2010 for new HIV-specific health status instruments. Results yielded four instruments that appeared to meet our inclusion criteria [37-40]. Three instruments were HRQL/QOL measures; the Missoula-Vitas Quality-of-Life Index developed to assess quality of life in advanced HIV illness in a palliative care setting [37], the Neurological Quality of Life Questionnaire, a general measure of quality of life in HIV infection [38], and the Chronic Illness Quality of Life Ladder developed to assess quality of life across four time periods (past, present, future, and life without a diagnosis of HIV) [39]. The fourth instrument was a lipodystrophy scale developed to assess the severity of lipodystrophy from the perspective of individuals living with HIV [40]. Similar to the instruments included in our study, none of these instruments were developed to assess the construct of disability. Also, none contained items that represent the dimension of uncertainty.

## Conclusions

No existing HIV-specific instrument fully captures the breadth and depth of disability experienced by adults living with HIV as conceptualized by the *Episodic Disability Framework*. Symptoms/impairments and difficulties carrying out day-to-day activities were characterized in greatest depth among most instruments, whereas challenges to social inclusion and uncertainty were less well represented. Nevertheless, these instruments may serve as a foundation from which to build a future instrument of disability. Future steps include using the *Episodic Disability Framework *as a foundation from which to establish a collection of items that will formulate a new instrument to describe disability experienced by adults living with HIV. Development of a new HIV disability questionnaire is currently underway.

## Competing interests

The authors declare that they have no competing interests.

## Authors' contributions

KO developed the research question, study design, performed the search strategy, reviewed instruments for inclusion, performed the document analysis, interpreted findings, and drafted the manuscript. This research was completed as part of KO's PhD thesis research study. AB and AD (co-supervisors) and CS and NY (committee members) participated in the development of the research question, study design, oversaw the analysis and helped to draft the manuscript. KK participated in the document analysis, interpretation of findings, and helped to draft the manuscript. All authors have read and approved the final manuscript.

## Supplementary Material

Additional file 1 Detailed Overview of Categories and Sub-Categories (and Codes) for the Document Analysis of Existing HIV-Specific Instruments Click here for file
