# Supplementary material for: How do existing HIV-specific instruments measure up? Evaluating the ability of instruments to describe disability experienced by adults living with HIV
Source: Health Qual Life Outcomes. 2010 Aug 19;8:88. doi: 10.1186/1477-7525-8-88 (PMC2936441; doi:10.1186/1477-7525-8-88)
Supplement: Additional file 1 — Detailed Overview of Categories and Sub-Categories (and Codes) for the Document Analysis of Existing HIV-Specific Instruments [file 1477-7525-8-88-S1.PDF]

Additional File 1 – Detailed Overview of Categories and Sub-Categories (and Codes) for the Document Analysis of Existing HIV-Specific Instruments

Episodic Disability Framework Components	Categories		Detailed Sub-Categories	Sub-Category Code (n=108)
**A. Dimensions of Disability**				
Episodic Disability *(n=72 detailed sub-categories)*	Symptoms / Impairments *(n=44 detailed sub-categories)*	Symptoms / Impairments Physical (SI) *(n=31 detailed sub-categories)*	fatigue / feeling tired / exhaustion / decreased energy level	SI-1
			diarrhea / bladder	SI-2
			nausea and vomit	SI-3
			headaches	SI-4
			rashes and skin sores, ulcers or discoloration	SI-5
			ulcers and upset stomach – GI symptoms	SI-6
			pins and needles & numbness and tingling (may or may not be secondary to neuropathy)	SI-7
			pain (aches and pains)	SI-8
			speech and swallowing problems	SI-9
			decreased libido	SI-10
			cardiovascular effects (secondary to stroke, myocardial infarction)	SI-11
			cardirespiratory impairments such as dyspnea, cough secondary to pneumonias	SI-12
			decreased endurance / activity tolerance	SI-13
			decreased balance (may or may not be secondary to stroke)	SI-14
			fever and sweats	SI-15
			weakness (may or may not be secondary to stroke or deconditioning)	SI-16
			cramps (muscle and stomach)	SI-17
			body composition changes (weight loss and gain may or may not be secondary to lipodystrophy)	SI-18
			decreased appetite	SI-19
			sleep loss / difficulty sleeping	SI-20
			hair loss	SI-21
			forgetfulness / difficulty focusing attention / making decisions / agitated	SI-22
			shaky hands / tremor*	SI-23
			change in handwriting*	SI-24
			bruising / bleeding*	SI-25
			dry mouth/mouth pain*	SI-26
			vision loss*	SI-27
			hearing loss*	SI-28
			dizziness*	SI-29
			swollen lymph or glands or feet*	SI-30
			genitals, menstruation, etc*	SI-31

Episodic Disability Framework Components	Categories		Detailed Sub-Categories	Sub-Category Code
Episodic Disability (continued) *(n=72 detailed sub-categories)*	Symptoms / Impairments (continued) *(n=44 detailed sub-categories)*	Symptoms / Impairments related to Stress, Anxiety, and Depression (SI-SAD) *(n=2 detailed sub-categories)*	stress, anxiety and depression (and nervous)	SI-SAD-1
			stress, anxiety and depression - suicide ideation*	SI-SAD-2
		Symptoms / Impairments related to Emotions (SI-E) *(n=11 detailed sub-categories)*	emotions (global)	SI-E
			fear	SI-E-1
			decreased self-esteem / confidence	SI-E-2
			shame /blame and embarrassment, guilt, regret and feeling alienated	SI-E-3
			loneliness	SI-E-4
			grief / bereavement*	SI-E-5
			adjustment difficulty*	SI-E-6
			feeling unsafe*	SI-E-7
			frustration / temper / agitated / anger / discouraged*	SI-E-8
			upset / distress / despair / hopelessness*	SI-E-9
			un-acceptance / not feeling loved*	SI-E-10
	Uncertainty *(n=2 detailed sub-categories)*	Uncertainty (UN)	worrying about the Future	UN-1
			impact of uncertainty on decision making	UN-2
	Difficulties with Day-to-Day Activities *(n=22 detailed sub-categories)*	Difficulties with Day-to-Day Activities (DAY)	difficulties with day-to-day activities (global)	DAY
			standing (indoors and outdoors)	DAY-1
			walking (indoors and outdoors)	DAY-2
			negotiating stairs	DAY-3
			transfers (moving from bed to chair, standing)	DAY-4
			dancing (and other leisure and recreational activities / vigorous activities e.g. exercise)	DAY-5
			activities of daily living –eating	DAY-6
			activities of daily living – bathing / hygiene	DAY-7
			activities of daily living – dressing	DAY-8
			household chores – cleaning	DAY-9
			household chores – dishes	DAY-10
			household chores – laundry	DAY-11
			household chores – cooking	DAY-12
			grocery shopping	DAY-13
			getting out paying bills (errands)	DAY-14
			attending doctor's appointments	DAY-15
			reaching*	DAY-16
			gripping*	DAY-17
			bending or lifting*	DAY-18
			sexual activity*	DAY-19
			balancing checkbook, paperwork for bills*	DAY-20
			driving*	DAY-21

Episodic Disability Framework Components	Categories		Detailed Sub-Categories	Sub-Category Code
	Challenges to Social Inclusion *(n=4 detailed sub-categories)*	Challenges to Social Inclusion (SOC)	parental roles	SOC-1
			work and school	SOC-2
			personal relationships (difficulty initiating or maintaining relationships either casual or intimate in nature with friends, family or partners	SOC-3
			difficulty engaging in other social / leisure activities (e.g. travel, joining the military)	SOC-4

B. Supplementary Content of Disability
(beyond dimensions of disability but within the *Episodic Disability Framework*)

Contextual Factors of Episodic Disability *(n=12 detailed sub-categories)*	Extrinsic Factors *(n=4 detailed sub-categories)*	Social Support (SUP)	level of support – finances / housing / insurance (environmental items)	SUP-1
			level of support – family / friends, etc.	SUP-2
			level of support – health care services, medications and personnel / transport	SUP-3
		Stigma (STIG)	stigma	STIG
	Intrinsic Factors *(n=8 detailed sub-categories)*	Living Strategies (STR)	living strategies – social interaction with others versus isolation / avoidance	STR-1
			living strategies – maintaining control – planning ahead, anticipating episodes, coping	STR-2
			living strategies – maintaining control – lifestyle	STR-3
			living strategies – attitudes and beliefs – outlook – hope & resiliency	STR-4
			living strategies –attitudes and beliefs – concerned about how will be remembered, breaking family line, destiny	STR-5
			living strategies-attitudes and beliefs – blocking HIV out of mind	STR-6
			living strategies – attitudes and beliefs – faith and spirituality	STR-7
		Personal Attributes (PER)	intrinsic factor-personal attributes (age, gender, ethnocultural background)	PER-1
Triggers of Episodes of Disability *(n=3 detailed sub-categories)*	Triggers of Disability *(n=3 detailed sub-categories)*	Triggers (TRIG)	trigger – serious illness / opportunistic infection occurrence	TRIG-1
			trigger – anti-retroviral medications	TRIG-2
			trigger – anti-retroviral medications – burden and impact on life	TRIG-3

Episodic Disability Framework Components	Categories		Detailed Sub-Categories	Sub-Category Code
**C. Extraneous Content Beyond the *Episodic Disability Framework***				
Other Topics Covered by Items Beyond the Episodic Disability Framework *(n=21 detailed sub-categories)*	Other *(n=21 detailed sub-categories)*	Other (O)	sexual behaviour*	O-1
			medications – changing / compliance*	O-2
			importance of activities / items*	O-3
			satisfaction / pleased*	O-4
			enjoyment and interest (of life, food, being with others, sex)*	O-5
			confusion (in relation to body appearance)*	O-6
			global health rating*	O-7
			global illness rating*	O-8
			quality of life*	O-9
			Do not resuscitate (DNR) item*	O-10
			global side effects of treatment*	O-11
			"other" open ended option*	O-12
			specific non-HIV related question*	O-13
			HIV-risk group question*	O-14
			implications of caring for someone else*	O-15
			types of medication taken*	O-16
			diagnostic tests / treatments taken / abnormal blood tests*	O-17
			number of days in hospital*	O-18
			number of days at home*	O-19
			number of days in bed*	O-20
			number of days cut down on things you usually do because of your health*	O-21

*Indicates new sub-categories derived from items in the existing instruments.
